# Prevalence of Risk of Sarcopenia in Polish Elderly Population—A Population Study

**DOI:** 10.3390/nu14173466

**Published:** 2022-08-24

**Authors:** Magdalena Milewska, Zuzanna Przekop, Dorota Szostak-Węgierek, Mariola Chrzanowska, Filip Raciborski, Iwona Traczyk, Beata Irena Sińska, Bolesław Samoliński

**Affiliations:** 1 Department of Clinical Dietetics, Faculty of Health and Science, Medical University of Warsaw, 01-445 Warszawa, Poland; 2 Department of Prevention of Environmental Hazards and Allergology, Faculty of Health Sciences, Medical University of Warsaw, 02-091 Warszawa, Poland; 3 Department of Econometrics and Statistics, Institute of Economics and Finance, Warsaw University of Life Sciences, 02-787 Warszawa, Poland; 4 Department of Human Nutrition, Faculty of Health Sciences, Medical University of Warsaw, 01-445 Warszawa, Poland

**Keywords:** sarcopenia, aged, frailty, obesity, risk factors

## Abstract

Sarcopenia in the elderly population is a public health challenge, and there are few data on its prevalence in Europe. In this study, we investigated the prevalence of sarcopenia in the elderly Polish population and its association with the level of obesity and co-existing diseases. We conducted a population-based cross-sectional study involving 823 men and 1177 women aged 65 years and older, randomly selected from the population living in the territory of the Republic of Poland between 2017 and 2020. We analyzed the results of body mass index (BMI), waist circumference (WC), waist-to-hip ratio (WHR), and waist-to-height ratio (WHtR). Risk of sarcopenia was assessed with the simple questionnaire to rapidly diagnose sarcopenia (SARC-F), and sarcopenic obesity risk was defined as the combination of anthropometry and SARC-F results. In addition, we collected disease data with an author questionnaire. The prevalence of risk of sarcopenia was 18.6% (22.3% in women and 13.2% in men), and its incidence significantly increased with age in both sexes. The risk of sarcopenic obesity was more common in women than in men, and it was higher in the older age group, except for sarcopenic obesity diagnosed by the WHR criteria. The group of elderly with concomitant diseases had a higher risk of developing sarcopenia, which emphasizes the need to monitor sarcopenia when concomitant diseases are diagnosed. In both groups, risk of sarcopenia was associated with motor and respiratory system diseases, type 2 diabetes, and neurological diseases. This study highlights that the risk of sarcopenia in the elderly population affects women to a greater extent than men. It is important to identify the elderly at risk of sarcopenia in routine clinical practice to develop long-term prevention strategies.

## 1. Introduction

Sarcopenia is considered a muscle disorder characterized by progressive loss of muscle strength and mass due to aging and/or chronic diseases [1]. While many studies have been published on sarcopenia in Asian populations, there are far fewer studies examining the prevalence and clinical outcomes in the elderly in Europe. Depending on the criteria used to diagnose sarcopenia, discrepancies in the prevalence of sarcopenia can be observed. It ranges from 5–13% in 60–70 years old to 11–50% in people older than 80 years [2] and up to 76% of acutely hospitalized elderly patients [3,4]. The etiology of sarcopenia is multifactorial. Although age is a major cause, other intrinsic and extrinsic factors are also considered as possible causes for the development of sarcopenia, such as physical inactivity, polypharmacy, vitamin D deficiency, poor nutritional status, and multimorbidity [5].

It is known that there is a significant association between sarcopenia and malnutrition in hospitalized older adults [6]. However, sarcopenia may also be associated with obesity and may contribute to poorer overall survival [7], higher risk of frailty [8], and higher risk of type 2 diabetes mellitus [9]. It is worth noting that a recent study confirmed that low appendicular skeletal mass index (ASMI) is common in community-dwelling elderly with normal nutritional status. Moreover, a positive association was found between BMI, calf circumference, bone mass, and ASMI [10]. These observations underscore the need for early screening for sarcopenia and provide the background for the development of national prevention strategies at the population level. Many studies indicate higher health care costs for sarcopenic patients compared with non-sarcopenic patients. However, the quality of research does not support a strong conclusion that sarcopenia increases the overall cost of care [11]. Given the high prevalence of sarcopenia in the elderly population and its association with poor outcomes, such as worsening functional status, disability, loss of independence, and increased risk of death, sarcopenia may be considered a geriatric syndrome [12,13]. Considering that sarcopenia is a public health challenge, we considered it important to conduct a detailed study on sarcopenia risk and its determinants in community-dwelling older adults. The aim of the study was to investigate the prevalence of sarcopenia risk in the older Polish population and its association with the level of obesity and co-existing diseases.

## 2. Materials and Methods

### 2.1. Design and Methods

This was a population-based cross-sectional study involving 823 men and 1177 women aged 65 years and older, randomly selected from the population living in the territory of the Republic of Poland. The research was carried out from 2017 to 2020. The design and methods of the study are presented in detail in a separate paper [14]. The study was approved by the Ethics Committee of the Medical University of Warsaw (AKBE/163/17 and AKBE164/17) and was conducted under the Ministry of Health Programme: National Health Programme for 2016–2020.

### 2.2. Data Collection and Instruments

Prior to the pandemic triggered by COVID-19 (October 2017–March 2020), all data were collected using the computer-assisted personal interviews (CAPI) method; thereafter the computer-assisted telephone interviews (CATI) method was used (June 2020–December 2020). An author questionnaire was used to collect demographic and clinical data.

#### 2.2.1. Anthropometric Measurements

Trained interviewers performed anthropometric measurements (height, body weight, and waist and hip circumferences) according to the recommendations of the WHO [15]. Body weight was measured with a portable electronic scale (Omron, model HBF-212, Omron Healthcare Ltd., Kyoto, Japan) with an accuracy of 0.1 kg [16]. For the measurement of body weight and height, respondents wore light clothing with empty pockets and no shoes. Body height was measured with an anthropometric tape measure and an angle measure by placing a sticky note on the wall above the tip of the respondent nose so that he or she touched the note with the back of his head. Then the angle was placed on the top of the respondent’s head at right angles to the wall. The interviewer marked a point with a pencil at the level of the lowest edge of the angle. The distance between the marked point and the floor was measured with an accuracy of 0.1 cm using an anthropometric tape measure. 

For waist circumference (WC) measurement, the respondents stood upright and relaxed. Measurements were taken after the subjects had taken several free breaths. The tape measure was placed parallel to the floor around the abdomen at the narrowest point (between the lowest rib and the top of the hip bone). The result was noted with an accuracy of 1 mm. Waist circumference categories were classified according to the IDF criteria [17].

To measure hip circumference, the interviewer placed the tape measure around the hip at the widest part of the buttocks, below the hip plates (directly above the pubic bone in women and between the iliac crest and the pubic bone in men). The tape was positioned parallel to the ground. The result was measured with an accuracy of 1 mm.

Body mass index (BMI) was calculated by dividing weight (kg) by height (m) squared. BMI categories were classified according to the CDC criteria [16] and the criteria established by Lipschitz for the elderly population, with a range of normal values of 22–27 kg/m^2^ [18].

Adipose tissue distribution was assessed using the waist-to-hip ratio (WHR), which is calculated by dividing waist circumference by hip circumference in centimeters. In women, values of 0.85 or greater indicate abdominal (visceral, central) obesity and less than 0.85 indicate gluteal (gynoid, peripheral) obesity. In men, the cutoff value is 0.9 [4]. The waist-to-height ratio (WHtR), waist circumference and body height ratio were calculated. A value of ≥0.5 indicates an increased risk of cardiovascular disease and diabetes in both men and women [19].

#### 2.2.2. Sarcopenia Risk Assessment

Patients at risk of sarcopenia were identified using the simple questionnaire to rapidly diagnose sarcopenia (SARC-F) developed by Malmstrom TK and Morley JE [20]. We used the Polish version of the SARC-F questionnaire validated in free-living elderly [21]. The instrument tests five domains: (1) strength, (2) assistance with walking, (3) getting up from a chair, (4) climbing stairs, and (5) falls. A SARC-F score value ≥ 4 was considered diagnostic.

Sarcopenic obesity risk was defined by the coexistence of sarcopenia risk (SARC-F score value ≥ 4) and obesity defined as BMI value ≥ 30 kg/m^2^ or abdominal obesity defined by one of the following criteria: WC ≥102 (M), ≥88 (F); WHR ≥ 0.9 (M), ≥0.85 (F); or WHtR > 0.5.

### 2.3. Data Analysis

The collected data were presented using descriptive statistics. For qualitative variables, the chi-square test was used. Quantitative variables were compared using the Student’s *t*-test and the Z-test (Tests About a Population Mean and About a Population Proportion). For all computations, a significance level of alpha equals 0.05 (*p* value < 0.05) used. Calculations were performed using R packages.

## 3. Results

### 3.1. Samples Characteristics

The demographic characteristics of all 2000 subjects studied, divided into sex and age groups, are shown in Table 1. The women were better educated, more often widowed, and in a worse financial situation than the men. The anthropometric characteristics of the subjects are shown in Table 2. Women had a higher mean BMI than men (28.00 ± 5.13 vs. 27.17 ± 3.77, respectively; *p* < 0.001), were more often obese according to the criteria set by the WHO (32.8% vs. 16.9%, respectively; *p* < 0.001), and were more often overweight according to the criteria set for the elderly population (53.6% vs. 47.1%, respectively; *p* < 0.001). Men had a higher mean waist circumference (94.31 ± 13.01 vs. 91.80 ± 13.78, respectively; *p* < 0.001) but a lower percentage of the highest category of WC values (25.7% vs. 60.9%, respectively; *p* < 0.001) than women. In contrast, abdominal obesity, defined by the WHR value, was more common in men (85.8% vs. 61.9%, respectively; *p* < 0.001). The frequency of WHtR values above 0.5 was higher in women than men (74.8% vs. 66.6%, respectively; *p* < 0.001).

The mean SARC-F score was higher in women than in men (2.08 ± 1.96 vs. 1.22 ± 1.72, respectively; *p* < 0.001). Sarcopenia risk, defined as SARC-F score ≥ 4, of the whole group was 18.6% and was more frequent in women (22.3% vs. 13.2%, respectively; *p* < 0.001). The frequency of sarcopenia significantly increased with age in both sexes.

### 3.2. Risk of Prevalence of Sarcopenia and Sarcopenic Obeisty in Adiposity Groups Determined by BMI, WC, WHR, and WHtR

Table 3 shows the prevalence of sarcopenia risk in adiposity groups. In both sexes, risk of sarcopenia occurred more frequently in the older age groups and tended to increase in almost every higher adiposity group by BMI category. In women, the risk of sarcopenia was higher than in men in almost every range of the WC and BMI categories. Both WHR and WHtR values appeared to be negatively associated with sarcopenia risk in men, while in women WHtR was positively related, and there was no relationship for WHR. The prevalence of sarcopenic obesity risk is shown in Table 4. The risk of sarcopenic obesity was more common in women than in men. The differences were highly statistically significant, with the exception of sarcopenia obesity risk, which was determined according to WHR criteria. In both sexes, the risk of sarcopenic obesity was significantly higher in the older age groups, except for sarcopenia obesity risk diagnosed using the WHR criteria.

### 3.3. Association between the Risk of Sarcopenia and Incidence of Diseases

Table 5 shows the diseases that occurred in sarcopenic individuals. In both groups, risk of sarcopenia was associated with diseases of the motor system, type 2 diabetes, respiratory system, and neurological diseases. In addition, risk of sarcopenia was associated with allergies, urinary tract diseases, cardiovascular diseases, and type 1 diabetes in women as opposed to men.

## 4. Discussion

It is commonly known that sarcopenia has profound health consequences, such as disability, increased dependence, and even death [12,13]. The exact prevalence of sarcopenia, especially in free-living elderly, is underdiagnosed due to discrepancies in the use of the European Working Group on Sarcopenia in Older People—EWGSOP1 or EWGSOP2 criteria [22,23] and low social awareness. Most studies regarding sarcopenia have been conducted in Asian populations. Yi-Wu et al. concluded that sarcopenia in Asians has several unique characteristics that differ from Caucasians due to differences in body composition (less muscle mass, lower handgrip strength, and more visceral adipose tissue), making it difficult to compare results [24]. The recent meta-analysis showed that the prevalence of sarcopenia was lower in Asian countries than in non-Asian countries [25]. There is a lack of data on older adults at risk for sarcopenia in non-Asian countries. This paper is one of the first studies describing the risk of sarcopenia among community-dwelling older people in Poland and its association with the level of obesity and co-existing diseases.

According to our analysis, the overall prevalence of those at risk of sarcopenia was 18.6%. Similar results were reported by Krzymińska-Siemaszko et al.; 10.4% to 33.0% of participants were identified as having an increased risk of sarcopenia, depending on the modified SARC-F questionnaire used. A trend toward more frequent occurrences of risk factors for sarcopenia in women than in men was noted; however, the group size of women did not allow comparative analysis [26]. In our study, the overall prevalence of elderly adults at risk of sarcopenia was higher in women than in men. Similar to our results, Peterman-Rocha et al. showed that both age and female sex were associated with a higher probability of sarcopenia [27]. Similar findings were observed by Yang et al. in which women had a 20% (age-adjusted OR = 1.20, 95% CI 0.98 to 1.47) higher risk of developing sarcopenia than men [28]. The currently available literature is inconclusive regarding sex differences in the prevalence of sarcopenia. Interestingly, in women, the risk of sarcopenia was higher in overweight and obese subjects than in subjects with normal body weight. In contrast to our observations, other authors reported that overweight in women (aged 38–73 years) was associated with a lower risk of sarcopenia [27,29]. However, in our study this association disappeared when the BMI criterion was used, which is recommended for the diagnosis of sarcopenia in the elderly. It should be noted that the risk of sarcopenic obesity was more prevalent in women than in men, with the exception of diagnosing sarcopenia, which was determined according to the WHR criteria. In a Chilean study, sarcopenia was also negatively associated with overweight and obesity diagnosed using BMI value [30]. A Brazilian study showed that older adults being at risk of malnutrition (assessed with the Mini Nutritional Assessment) had a higher risk of sarcopenia (OR = 13.6; 95% CI: 1.55–11.38; *p* < 0.05), while a BMI ≥ 27 kg/m^2^ was a protective factor (OR = 0.02; 95% CI: 0–0.06; *p* < 0.001) [31]. Previous studies have also shown that malnourished elderly are at risk of sarcopenia [32,33,34]. In an elderly Iranian population, increased body fat mass was identified as a risk factor for sarcopenia [35], which is partly consistent with our findings. This could be due to sarcopenic obese individuals tending to accumulate fat in muscles, which could negatively affect skeletal muscle function through increased levels of proinflammatory cytokines [36]. It is well documented that ageing is associated with negative changes in body composition, which often occur independently of changes in body weight. In particular, it is observed that the decline in muscle mass accelerates in the eighth decade of life. However, most striking, is the redistribution of fat mass to the abdominal region and its storage in type 2 muscle fibers, which affects muscle quality. These changes may be accelerated by chronic diseases [37].

Studies in smaller groups, mainly from clinical trials, suggest that sarcopenia is more common in malnourished people when more advanced diagnostic methods are used [38]. Sarcopenia is less common in obese people, which may partly explain the obesity paradox phenomenon, i.e., that the risk of dying from cardiovascular disease is lower in obese patients than in undernourished patients [38]. However, this applies to obesity assessed using BMI, which includes fat mass and fat-free mass. This phenomenon does not occur with other measures of obesity, such as waist circumference. It would be interesting to review the prevalence of sarcopenia at the population level in different groups in relation to the degree of body fat, which was also highlighted by Dufour et al. [39].

Elderly people are at risk of developing age-related diseases, i.e., the coexistence of two or more diseases [40]. It is estimated that more than 50% of the elderly suffer from three or more chronic diseases [41]. It has been demonstrated that individuals aged 40–70 years with multimorbidity are almost twice as likely to have sarcopenia compared with healthy participants [OR 1.96 (95% CI: 1.91, 2.02)] [42]. Pacificio et al. reported that the prevalence of sarcopenia in individuals with CVD was 31.4% (95% CI: 22.4–42.1%), with dementia—26.4% (95% CI: 13.6–44.8%), with diabetes mellitus—31.1% (95% CI: 19.8–45.2), and with respiratory diseases 26.8% (95% CI: 17.8–38.1%) [43]. Due to multiple chronic diseases, polypharmacy (taking at least five medications) is now common among older adults. Its prevalence ranges from 27.0% to 59.0% in community-dwelling older adults and has an unfavorable impact on functional status, fall risk, and frailty [44,45,46].

We have shown that sarcopenia was more common in men and women who had musculoskeletal and respiratory diseases, type 2 diabetes, and neurological diseases as well as in women with allergies, urinary tract diseases, cardiovascular diseases, and type 1 diabetes. It is commonly known that sarcopenia shares risk factors with cardiovascular disease, diabetes mellitus, respiratory diseases, and dementia [43]. Our results confirmed the association of sarcopenia risk with cardiovascular disease and diabetes mellitus type 1, but only in women. On the one hand, the prevalence of sarcopenia is increased in patients with chronic heart failure, which contributes to reduced physical capacity and frailty [47]. On the other hand, elderly with sarcopenia had higher levels of hs-CRP and a higher risk of arteriosclerosis. Additionally, serum hs-CRP level was an independent risk factor for sarcopenia [48]. Metabolic syndrome has been associated with poorer muscle quality, especially in obese and overweight adults [49]. There is a lack of data to identify the elderly population at risk of sarcopenia in particular diseases in community-dwelling settings.

The prevalence of sarcopenia in patients with type 2 diabetes mellitus has not been fully elucidated. However, there is a decrease in muscle mass associated with insulin resistance and a chronic inflammatory state. It is worth emphasizing the co-occurrence of obesity, type 2 diabetes mellitus, and sarcopenia. Independently, low muscle mass and excess adipose tissue have been associated with a higher incidence of metabolic disorders [50,51]. In a Brazilian study, it was reported that diabetic patients had low handgrip strength and altered bone mass density [52].

Our findings confirmed a higher prevalence of sarcopenia risk in both females and males who suffered from diseases associated with motor and respiratory systems, neurological disease, and type 2 diabetes. In neurological and motor system disease, many factors contribute to the risk of sarcopenia. These include decreased physical activity, chronic inflammation, and pain [53]. Peterman and colleagues reported that sarcopenia was more prevalent in those with rheumatoid arthritis. Torri et al. concluded that longer disease duration, joint degradation, and malnutrition were positively associated with sarcopenia [54]. In another study, sarcopenia was related to age and bone erosion, but not to rheumatoid disease activity [55]. One of the studies showed that osteoporosis was significantly associated with a diagnosis of sarcopenia within 4 years (OR, 2.99; 95% CI, 1.46–6.12; *p* < 0.01) [56].

Interestingly, we found an increased risk of sarcopenia in women with allergies and urinary tract disease. Several mechanisms may affect muscle mass and function in chronic kidney disease, such as low protein intake, physical inactivity, inflammation, and low serum vitamin D concentration [57]. Moreover, chronic kidney disease is a catabolic state, associated with increased synthesis of inflammatory markers and a significant decline in muscle synthesis [58,59]. In the present study, we could not specify particular diseases that increase the risk of sarcopenia because we classified different diseases into groups. The interplay between sarcopenia and chronic diseases in elderly patients requires further study to clarify the impact in terms of long-term implications. Considering that sarcopenia is underdiagnosed in clinical practice [4], identifying the population at risk of sarcopenia is of paramount importance to introduce better management of comorbid diseases.

## 5. Limitations

Our study has some limitations. First, the COVID-19 pandemic forced changes in the research methodology. Second, we used the SARC-F questionnaire, as a highly specific screening tool recommended by EWGSOP2 but which is highly criticized in recent publications for its low sensitivity [60,61,62]. However, the use of more advanced methods, such as bioimpedance, was not feasible at the population level.

Thirdly, the character of our study made it difficult to determine specific conditions for sarcopenia risk. Fourth, our exclusively Caucasian study population limits the generalizability of our results to other populations. To our knowledge, a strength of our analysis is that this is one of the first studies to examine the prevalence risk of sarcopenia in community-dwelling older adults.

## 6. Conclusions

This study has shown that the risk of sarcopenia in the elderly population affects women to a greater extent than men. The group of elderly with concomitant diseases has a higher risk of developing sarcopenia, which emphasizes the need to monitor sarcopenia when concomitant diseases are diagnosed. Our results confirmed a higher prevalence of sarcopenia risk in both women and men who had motor and respiratory system diseases, type 2 diabetes, or neurologic diseases. In addition, women, but not men, who had urinary tract disease, allergies, cardiovascular disease, and type 1 diabetes had an increased risk of sarcopenia. It is important to identify the elderly at risk for sarcopenia and sarcopenic obesity in routine clinical practice to develop long-term prevention strategies. Further studies are needed to better understand population-level risk factors of sarcopenia.

## Figures and Tables

**Table 1:** Demographic characteristics of the study group, divided into age groups, *n*(%).

Characteristic		Males, Years of Age				Females, Years of Age				*p* for Difference between Genders
		Total ≥ 65 (*n* = 823)	65–74 (*n* = 548)	≥75 (*n* = 275)	*p*	Total ≥ 65 (*n* = 1177)	65–74 (*n* = 633)	≥75 (*n* = 544)	*p*	
**Education**	Primary/middle (gymnasium), *n*(%)	129 (15.7)	66 (12.0)	63 (22.9)	<0.001	193 (16.4)	52 (8.2)	141 (25.9)	<0.001	<0.001
	Middle (basic vocational school), *n*(%)	441 (53.6)	313 (57.1)	128 (46.5)		489 (41.5)	277 (43.8)	212 (39.0)		
	Secondary (general or technical), *n*(%)	198 (24.1)	139 (25.4)	59 (21.5)		417 (35.4)	267 (42.2)	150 (27.6)		
	Tertiary (Bachelor’s degree programmes, Master degree programmes), *n*(%)	55 (6.7)	30 (5.5)	25 (9.1)		78 (6.6)	37 (5.8)	41 (7.5)		
**Marital status**	Single, *n*(%)	18 (2.2)	17 (3.1)	1 (0.4)	<0.001	17 (1.4)	11 (1.7)	6 (1.1)	<0.001	<0.001
	In relationship, *n*(%)	654 (79.5)	467 (85.2)	187 (68.0)		692 (58.8)	426 (67.3)	266 (48.9)		
	Divorced/separated, *n*(%)	34 (4.1)	16 (2.9)	18 (6.5)		49 (4.2)	33 (5.2)	16 (2.9)		
	Widowed, *n*(%)	117 (14.2)	48 (8.8)	69 (25.1)		419 (35.6)	163 (25.8)	256 (47.1)		
**Financial condition**	We are wealthy, we do not have to save even for larger expenses, *n*(%)	4 (0.5)	2 (0.4)	2 (0.7)	0.083	0	0	0	0.14	<0.001
	We have enough money for all expenses, and we can save some money, *n*(%)	106 (12.9)	63 (11.5)	43 (15.6)		88 (7.5)	47 (7.4)	41 (7.5)		
	We have enough money for everyday expenses, but we cannot afford more, *n*(%)	618 (75.1)	428 (78.1)	190 (69.1)		896 (76.1)	493 (77.9)	403 (74.1)		
	We have to deny ourselves many things so that we have enough money to live, *n*(%)	90 (10.9)	52 (9.5)	38 (13.8)		185 (15.7)	87 (13.7)	98 (18.0)		
	We don’t have enough money, even for the most urgent needs, *n*(%)	5 (0.6)	3 (0.5)	2 (0.7)		8 (0.7)	6 (0.9)	2 (0.4)		

**Table 2:** Anthropometric characteristics and prevalence of sarcopenia risk diagnosed according to the SARC-F questionnaire in the study group, divided into age groups.

Characteristic		Males, Years of Age				Females, Years of Age				*p* for Difference between Genders
		Total ≥ 65 (*n* = 823)	65–74 (*n* = 548)	≥75 (*n* = 275)	*p*	Total ≥ 65 (*n* = 1177)	65–74 (*n* = 633)	≥75 (*n* = 544)	*p*	
**Body mass (kg)**	Mean ± SD	81.98 ± 12.5	82.49 ± 12.37	80.98 ± 12.71	0.18	74.63 ± 13.79	75.26 ± 13.90	73.89 ± 13.63	0.53	<0.001
	Med.	81.4	82.0	80.2		74.0	74.0	74.0		
	Min–max	48–130	49–130	48–121		35.3–140	43.6–140	35.3–118		
**BMI (kg/m^2^)**	Mean ± SD	27.17 ± 3.77	27.22 ± 3.8	27.07 ± 3.72	0.98	28.00 ± 5.13	28.02 ± 5.12	27.97 ± 5.14	0.07	<0.001
	Med.	26.71	26.83	26.61		27.39	27.31	28.68		
	Min–max	16.81–47.75	16.81–47.75	17.63–46.09		16.05–45.39	17.58–45.39	16.05–44.96		
**BMI (kg/m^2^), ranges, according to WHO, *n*(%)**	<18.5	3 (0.4)	2 (0.4)	1 (0.4)	0.89	10 (0.8)	7 (1.1)	3 (0.6)	0.02	<0.001
	18.5–24.9	239 (29.0)	154 (28.1)	85 (30.9)		371 (31.5)	195 (30.8)	176 (32.4)		
	25.0–29.9	442 (53.7)	299 (54.6)	143 (52.0)		410 (34.8)	237 (37.4)	173 (31.8)		
	≥30	139 (16.9)	93 (17.0)	46 (16.7)		386 (32.8)	194 (30.6)	192 (35.3)		
**BMI (kg/m^2^), ranges for elderly, *n*(%)**	<22	32 (3.9)	22 (4.0)	10 (3.6)	0.73	127 (10.8)	64 (10.1)	63 (11.6)	0.72	<0.001
	22.0–26.9	403 (49.0)	263 (48.0)	140 (50.9)		419 (35.6)	228 (36.0)	191 (35.1)		
	≥27.0	388 (47.1)	263 (48.0)	125 (45.5)		631 (53.6)	341 (53.9)	290 (53.3)		
**WC (cm)**	Mean ± SD	94.31 ± 13.01	93.70 ± 12.73	95.72 ± 13.57	0.04	91.80 ± 13.78	91.77 ± 13.42	91.85 ± 14.27	0.92	<0.001
	Med.	91	90	93		90.2	90	91.1		
	Min–max	69–177	69–177	70–145		57–159	59.9–159	57–155		
**WC (cm), ranges, *n*(%)**	<94 (M); <80 (F)	439 (57.1)	318 (59.2)	121 (52.2)	0.11	183 (17)	94 (15.1)	89 (19.5)	0.07	<0.001
	94–102 (M); 80–88 (F)	132 (17.2)	92 (17.1)	40 (17.2)		239 (22.2)	157 (25.2)	82 (18.0)		
	≥102 (M); ≥88 (F)	198 (25.7)	127 (23.6)	71 (30.6)		657 (60.9)	372 (59.7)	285 (62.5)		
**WHR**	Mean ± SD	0.97 ± 0.09	0.96 ± 0.08	0.98 ± 0.09	0.001	0.87 ± 0.09	0.87 ± 0.09	0.86 ± 0.09	0.06	<0.001
	Med.	0.95	0.95	0.97		0.87	0.87	0.87		
	Min–max	0.56–1.99	0.56–1.99	0.7–1.33		0.48–1.69	0.48–1.69	0.51–1.29		
**WHR, ranges, *n*(%)**	<0.9 (M); <0.85 (F)	109 (14.2)	72 (13.4)	37 (15.9)	0.37	411 (38.1)	228 (36.6)	183 (40.1)	0.25	<0.001
	≥0.9 (M); ≥0.85 (F)	660 (85.8)	465 (86.6)	195 (84.1)		668 (61.9)	395 (63.4)	273 (59.9)		
**WHtR**	Mean ± SD	0.54 ± 0.07	0.54 ± 0.07	0.55 ± 0.08	0.001	0.56 ± 0.09	0.56 ± 0.09	0.57 ± 0.09	0.06	<0.001
	Med.	0.53	0.52	0.54		0.56	0.55	0.56		
	Min–max	0.4–1.00	0.4–1.00	0.41–0.81		0.35–1	0.35–1	0.35–1		
**WHtR, ranges, *n*(%)**	≤ 0.5	257 (33.4)	199 (37.1)	58 (25.0)	0.01	272 (25.2)	163 (26.2)	109 (23.9)	0.44	<0.001
	>0.5	512 (66.6)	338 (62.9)	174 (75.0)		807 (74.8)	460 (73.8)	347 (76.1)		
**SARC-F, score**	Mean ± SD	1.22 ± 1.72	0.79 ± 1.36	2.08 ± 2.02	<0.001	2.08 ± 1.96	1.6 ± 1.73	2.63 ± 2.06	<0.001	<0.001
	Med.	0	0	1		2	1	2		
	Min–max	0–10	0–7	0–10		0–9	0–9	0–9		
**Sarcopenia, *n*(%)**	No (SARC-F score 0–3)	714 (86.8)	509 (92.9)	205 (74.5)	<0.001	914 (77.7)	548 (85.0)	366 (67.3)	<0.001	<0.001
	Yes (SARC-F score ≥ 4)	109 (13.2)	39 (7.1)	70 (25.5)		263 (22.3)	85 (13.4)	178 (32.7)		

BMI—body mass index; WC—waist circumference; WHR—waist-to-hip ratio; WHtR—waist-to-height ratio; SARC-F—simple questionnaire to rapidly diagnose sarcopenia.

**Table 3:** Sarcopenia risk prevalence in adiposity groups.

Characteristics		Males, Years of Age			Females, Years of Age			*p* for Difference between Genders
		Total (*n* = 823)	65–74 (*n* = 548)	≥75 (*n* = 275)	Total (*n* = 1177)	65–74 (*n* = 633)	≥75 (*n* = 544)	
**BMI (kg/m^2^), ranges, according to WHO** ***n*(%)**	<18.5	1 (33.3%)	1 (50.0%)	0 (0.0%)	3 (30.0%)	2 (28.6%)	1 (33.3%)	<0.001
	18.5–24.9	28 (11.7%)	9 (5.8%)	19 (22.4%)	62 (16.7%)	15 (7.7%)	47 (26.7%)	
	25.0–29.9	60 (13.6%)	23 (7.7%)	37 (25.9%)	82 (20.0%)	32 (13.5%)	50 (28.9%)	
	≥30	20 (14.4%)	6 (6.5%)	14 (30.4%)	116 (30.1%)	36 (18.6%)	80 (41.7%)	
**BMI (kg/m^2^), ranges for elderly** ***n*(%)**	<22	2 (6.3%)	1 (4.5%)	1 (10.0%)	17 (13.4%)	4 (6.3%)	13 (20.6%)	<0.001
	22.0–26.9	56 (13.9%)	22 (8.4%)	34 (24.3%)	84 (20.0%)	26 (11.4%)	58 (30.4%)	
	≥27.0	51 (13.1%)	16 (6.1%)	35 (28.0%)	162 (25.7%)	55 (16.1%)	107 (36.9%)	
**WC (cm), ranges** ***n*(%)**	No data	8 (14.8%)	2 (18.2%)	6 (14.0%)	14 (14.3%)	0 (0.0%)	14 (15.9%)	<0.001
	<94 (M); <80 (F)	60 (13.7%)	24 (7.5%)	36 (29.8%)	38 (20.8%)	11 (11.7%)	27 (30.3%)	
	94–102 (M); 80–88 (F)	10 (7.6%)	6 (6.5%)	4 (10.0%)	45 (18.8%)	18 (11.5%)	27 (32.9%)	
	≥102 (M); ≥88 (F)	31 (15.7%)	7 (5.5%)	24 (33.8%)	166 (25.3%)	56 (15.1%)	110 (32.6%)	
**WHR, ranges** ***n*(%)**	No data	8 (14.8%)	2 (18.2%)	6 (14.0%)	14 (14.3%)	0 (0.0%)	14 (15.9%)	0.02
	<0.9 (M); <0.85 (F)	25 (22.9%)	9 (12.5%)	16 (43.2%)	94 (22.9%)	31 (13.6%)	63 (34.4%)	
	≥0.9 (M); ≥0.85 (F)	76 (11.5%)	28 (6.0%)	48 (24.6%)	155 (23.2%)	54 (13.7%)	105 (37.0%)	
**WHtR, ranges** ***n*(%)**	No data	8 (14.8%)	2 (18.2%)	6 (14.0%)	14 (14.3%)	0 (0.0%)	14 (15.9%)	<0.001
	≤0.5	38 (14.8%)	18 (9.0%)	20 (34.5%)	46 (16.9%)	16 (9.8%)	30 (27.5%)	
	>0.5	63 (12.3%)	19 (5.6%)	44 (25.3%)	203 (25.2%)	69 (15.0%)	134 (38.6%)	

BMI—body mass index; WC—waist circumference; WHR—waist-to-hip ratio; WHtR—waist-to-height ratio.

**Table 4:** Sarcopenic obesity risk prevalence.

Diagnostic Criteria	Males, Years of Age				Females, Years of Age				*p* for Difference between Genders
	Total ≥ 65 (*n* = 823)	65–74 (*n* = 548)	≥75 (*n* = 275)	*p*	Total ≥ 65 (*n* = 1177)	65–74 (*n* = 633)	≥75 (*n* = 544)	*p*	
**BMI ≥ 30 kg/m^2^** **and SARC-F score ≥ 4 *n*(%)**	20 (2.4)	6 (1.1)	14 (5.1)	0.000	116 (9.9)	36 (5.7)	80 (14.7)	0.000	<0.001
**WC ≥ 102 (M); ≥88 (F)** **and SARC-F score ≥ 4 *n*(%)**	31 (3.8)	7 (1.3)	24 (8.7)	0.000	166 (14.1)	56 (8.8)	110 (20.2)	0.000	<0.001
**WHR ≥ 0.9 (M); ≥0.85 (F) and SARC-F score ≥ 4 *n*(%)**	76 (9.2)	28 (5.1)	48 (17.5)	0.407	155 (13.2)	54 (8.5)	101 (18.6)	0.41	<0.12
**WHtR > 0.5** **and SARC-F score ≥ 4 *n*(%)**	63 (7.7)	19 (3.5)	44 (16.0)	0.000	203 (17.2)	69 (10.9)	134 (24.6)	0.000	<0.001

BMI—body mass index; WC—waist circumference; WHR—waist-hip ratio; WHtR—waist to height ratio; SARC-F—simple questionnaire to rapidly diagnose sarcopenia.

**Table 5:** Diseases present in sarcopenic subjects.

Group of Diseases	Males *n* = 109		Females *n* = 263	
	OR (CI)	*p*	OR (CI)	*p*
Allergies	1.01 (0.11–4.55)	ns	1.95 (1.04–3.58)	0.028
Diseases of urinary tract	1.58 (0.68–3.31)	ns	1.76 (0.97–3.09)	0.045
Cardiovascular diseases	1.32 (0.83–2.07)	ns	2.1 (1.57–2.82)	<0.001
Motoric system diseases	3.65 (2.28–5.8)	<0.001	3.36 (2.44–4.6)	0.000
Diabetes type 1	1.01 (0.11–4.55)	ns	2.21 (1.0–4.7)	0.035
Diabetes type 2	2.38 (1.0–5.22)	0.028	1.28 (1.28–3.19)	0.0017
Respiratory system diseases	3.31 (1.52–6.88)	0.0013	2.15 (1.2–3.77)	0.0071
Neurological diseases	13.17 (4.35–44.38)	<0.001	6.93 (2.88–17.88)	<0.001

ns—not significant.

## Data Availability

The data presented in the analysis are the property of the PolishMinistry of Health. The authors of the article, as the researchers responsible for the study, used themon the basis of the separate agreement with Ministry of Health.
